# Performance of MTBDR*plus* assay in detecting multidrug resistant tuberculosis at hospital level

**DOI:** 10.1186/s13104-017-2989-7

**Published:** 2017-12-01

**Authors:** Abebaw Kebede, Daniel Demisse, Meazashwork Assefa, Zerihun Getachew, Bazezew Yenew, Yared Tedla, Gobena Ameni

**Affiliations:** 1 Ethiopian Public Health Institute (EPHI), P.O.Box 1242, Addis Ababa, Ethiopia; 2 Aklilu Lemma Institute of Pathology (ALIP), Addis Ababa University (AAU), P.O.Box 1176, Addis Ababa, Ethiopia; 3 St. Peter TB Specialized Hospital, P.O.Box 21494, Addis Ababa, Ethiopia

**Keywords:** MTBDR*plus* assay, Multidrug-resistant tuberculosis, Mutation

## Abstract

**Objective:**

Multidrug-resistant tuberculosis (MDR-TB) case finding progressively increased in Ethiopia mainly as a result of the utilization of World Health Organization (WHO)-endorsed rapid technologies including MTBDR*plus* assay. However, there is inadequate data on routine testing performance of the MTBDR*plus* assay. Consequently, the aim of the study was to assess the routine performance of the MTBDR*plus* assay in detecting MDR-TB at St. Peter’s TB Specialized Hospital.

**Results:**

The sensitivity and specificity of MTBDR*plus* in detecting isoniazid (INH) resistance were 96.3 and 100%, respectively. While for rifampicin (RIF) 100% was recorded for both. Similarly, a sensitivity of 97.96% and a specificity of 100% was measured for detecting MDR-TB. Among 49 MTBDR*plus* RIF resistant isolates, 46 (93.9%) strains had *rpoB* mutation. S531L was the most common *rpoB* mutant (81.6% of RIF resistant cases). All MTBDR*plus* INH resistant isolates (n = 52) had S315T1 *katG* mutation.

## Introduction

Tuberculosis (TB) continues to be a major public health problem in Ethiopia. The country ranks seventh among the 22 high burden countries. According to the first population-based TB prevalence survey of Ethiopia, the estimated prevalence of smear positive and bacteriologically confirmed TB to be 108/100,000 and 277/100,000, respectively [1]. Moreover, the emergence of MDR-TB (resistance to at least isoniazid and rifampicin) largely affects the TB control program in the country. The estimated MDR-TB prevalence among the new and previously treated TB patients was 1.6 and 12%, respectively [2]. The development of MDR-TB can be caused by inadequate treatment, and the risk is higher in patients with the history of treatment failure and inappropriate treatment regimens [3]. Treatment based on drug resistance profiles ensures adequate treatment of the patients.

In the year 2014, 503 MDR-TB cases (39% of the estimated MDR-TB among the notified pulmonary TB cases) were reported from Ethiopia [2]. MDR-TB case finding progressively increased year-to-year as a result of the utilization of WHO-endorsed rapid molecular assays; MTBDR*plus* assay and Xpert MTB/RIF assay. However, still more than half of the estimated MDR-TB cases remain undetected in the population. Accurate and rapid detection of MDR-TB cases benefit the patients to receive treatment with second-line regimens and consequently cuts the transmission. In contrary, inaccurately classifying TB patients as MDR-TB patients due to diagnostic test limitation unnecessarily exposes patients for second-line anti-TB drugs for the prolonged period; often 18–24 months. These drugs are highly associated with drug side effects relative to the first-line anti-TB drugs [4].

Until 2010, the National TB Reference Laboratory (NRL) of Ethiopian Public Health Institute (EPHI) was the only TB culture and drug susceptibility testing (DST) laboratory that served the entire population of Ethiopia. Later on, eight more TB culture laboratories had been established at the center and in regions. In 2008, WHO endorsed Genotype^®^ MTBDR*plus* assay for rapid screening of patients at risk of MDR-TB [5]. This molecular line probe assay identifies *M. tuberculosis* complex (MTBC) and detects mutations that confer resistance to rifampicin (RIF) and isoniazid (INH). Mutations in *rpoB* gene (encodes RNA polymerase *b*-subunit) infer RIF resistance. Mutation in *katG* (encodes catalase peroxidase) and *inhA* (encodes enoyl-acyl carrier protein reductase) genes infer high- and low-level resistance to INH, respectively [6]. The national diagnostic algorithm suggests to use the assay independently or parallel with phenotypic DST [7]. The test can be applied directly to smear-positive samples and indirectly in smear-negative samples [7]. The MTBDR*plus* assay has been utilized for rapid DST in all newly established TB culture laboratories with no phenotypic DST option including St. Peter’s TB Specialized Hospital Laboratory. The assay is evaluated in many geographic locations of Africa [8–12]; however, there is limited documented evidence on the diagnostic accuracy of the MTBDR*plus* assay in detecting MDR-TB in Ethiopia. Therefore, we assessed the routine performance of MTBDR*plus* assay for detection of MDR-TB at St. Peter’s TB Specialized Hospital Laboratory.

## Main text

### Materials and methods

#### Setting

The study was conducted at St. Peter’s TB Specialized Hospital. Since 1961 Gregorian calendar the hospital has been serving as a referral point throughout the country for the management of highly complicated TB cases particularly MDR-TB. Moreover, the hospital is the first health facility to start MDR-TB treatment in Ethiopia with its own biosafety level (BSL)-3 TB culture and DST laboratory.

#### Clinical isolates

**Fig. 1:**
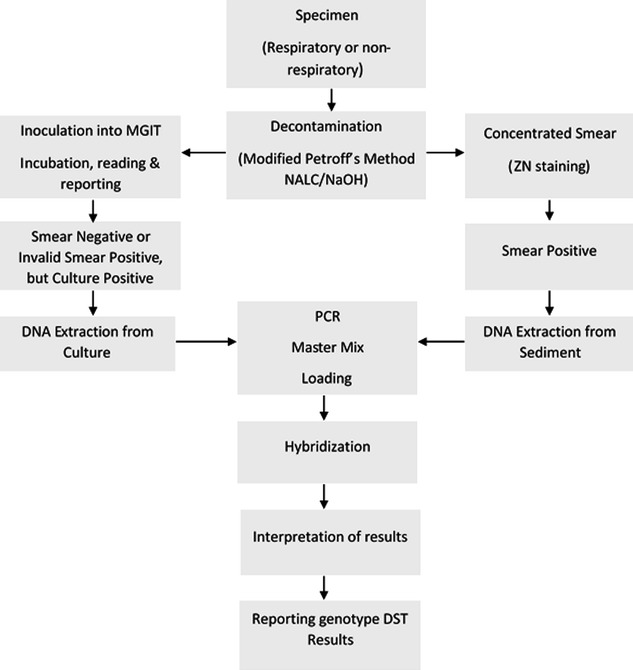
Routine drug-resistant TB laboratory diagnosis algorithm at St. Peter TB Specialized Hospital

Archived 72 mycobacterial clinical isolates from presumptive MDR-TB patients were utilized for phenotypic drug resistance analysis. The isolates were initially obtained from previously treated pulmonary specimens (sputum). Most isolates were from patients in Addis Ababa (60.5%) and the remaining from different regions of Ethiopia. The isolates were characterized for rifampicin (RIF) and isoniazid (INH) susceptibility using Genotype^®^ MTBDR*plus* assay (Hain Lifescience, Nehren, Germany) through the routine MDR-TB diagnostic service at St. Peter’s TB Specialized Hospital Laboratory during the year 2011/12 (Fig. 1). Forty-eight (66.7%) were found to be MDR-TB by MTBDR*plus* assay. Patients had received appropriate treatment completely relying on the test results of Genotype^®^ MTBDR*plus* assay with no phenotypic DST confirmation. The isolates were kept frozen in BACTEC™ MGIT™ 960 Tubes (BD, Sparks, MD, USA) at − 80 °C. Thawed liquid isolates (100 µl) was inoculated onto duplicate slant Lowenstein Jensen (LJ) media and incubated at 37 °C while waiting for confluent growth. The resultant mycobacterial growth was used for *M. tuberculosis* identification test and phenotypic DST.

#### Identification of *Mycobacterium tuberculosis* complex and *M. tuberculosis*

As described by Kumar et al. [13], the SD BIOLINE TB Ag MPT64 Rapid (Standard Diagnostics, Inc., Korea) was used for identification of *M. tuberculosis* complex (MTBC). For further confirmation of *M. tuberculosis*, Region-of-difference 9 (RD9) typing was performed as described by Parsons et al. [14] at Aklilu Lemma Institute of Pathobiology (ALIP).

#### Phenotypic drug susceptibility testing (DST)

Isolates identified as *M. tuberculosis* were analyzed using phenotypic conventional DST for RIF and INH resistance as described by Canetti et al. [15]. LJ based proportion method was used at critical concentrations (CC) 0.2 and 40 g/ml for INH and RIF, respectively. Final DST reading was taken after 6 weeks of incubation at 37 °C. The isolate was considered resistant if the proportion resistant *Bacilli* was higher than 1%. The phenotypic DST procedures were carried out, without the knowledge of MTBDR*plus* test results, at TB National Reference Laboratory (NRL) of Ethiopian Public Health Institute (EPHI).

#### MTBDR*plus* testing

Genotype^®^ MTBDR*plus* assay was carried directly from the specimen or indirectly from culture depending on the smear positivity of the pellet obtained from the processed specimen at St. Peter’s TB Specialized Hospital Laboratory following manufacturer’s instructions (Hain Lifescience). The procedure involved DNA extraction, multiplex amplification of target sequences with biotinylated primers, and DNA reverse hybridization. The test result was interpreted by considering the presence or absence of wild-type (WT) and mutant band on a strip. Absence of WT band or mutant band presence was an indication of resistance to an associated drug.

#### Quality control

*Mycobacterium tuberculosis* H37Rv (ATCC 27294) and *Mycobacterium bovis* (AF 2122/97) strains were used as positive controls. Molecular grade water was used as negative control for deletion typing. *M. tuberculosis* H37Rv was also included in every batch of ICA and phenotypic DST.

#### Data analysis

The sensitivity and specificity were calculated for assessing the routine performance of the MTBDR*plus* assay in comparing against the phenotypic DST. All the statistical analysis were performed using SPSS 20.0 Software (Statistical Package for the Social Sciences, Inc, Chicago, II, USA).

### Results

#### Identification of *Mycobacterium tuberculosis* complex and *M. tuberculosis*

All of the Mycobacterial isolates were identified as MTBC using ICA and further confirmed to be *M. tuberculosis* (MTB) by deletion typing. The MTB identification performance of MTBDR*plus* assay was 100% in comparing against ICA and deletion typing. There was no *M. bovis* identified among the clinical isolates by deletion typing (Data not shown).

#### Performance MTBDR*plus* assay in detecting RIF and INH resistance and MDR-TB against phenotypic DST

Forty-nine (68.1%) clinical isolates were identified as MDR-TB by the gold standard phenotypic DST; however, 48 (66.7%) of them were MDR-TB using MTBDR*plus* assay. All MTBDR*plus* RIF resistant isolates were found to be resistant by the phenotypic DST. We had two INH resistant discordant isolates in comparing against the standard method. The isolates were reported as INH susceptible by MTBDR*plus* while INH resistant by phenotypic DST i.e. one isolate was INH resistant by phenotypic DST but susceptible by MTBDR*plus* assay and the second isolate was MDR by phenotypic DST but it was RIF resistant only by MTBDR*plus* assay (Table 1).

**Table 1:** Performance MTBDR*plus* assay in detecting RIF resistance, INH resistance and MDR-TB against phenotypic DST

Drug resistance	LJ R	LJ S	LPA R	LPA S	Sensitivity, % (95% CI)	Specificity, % (95% CI)	PPV, % (95% CI)	NPV, % (95% CI)
MDR (RIF&INH)	49	23	48	24	97.96 (89.15–99.95)	100 (85.18–100.00)	100 (92.60–100.00)	95.83 (78.88–99.89)
INH	54	18	52	20	96.3 (87.25–99.55)	100 (81.47–100.00)	100 (93.15–100.00)	90 (68.3–98.77)
RIF	49	23	49	23	100 (92.72–100.00)	100 (85.18–100.00)	100 (92.72–100.00)	100 (85.18–100.00)

*LJ* Lowenstein Jensen DST, *LPA* line probe assay DST, *R* resistant, *S* sensitive, *CI* confidence interval

#### Mutations associated with rifampicin and isoniazid resistance

Among the 49 MTBDR*plus* RIF resistant isolates, 46 of them had *rpoB* mutation. The predominant *rpoB* mutant was S531L (81.6% of RIF resistant cases). H526Y (8.16%) and D516V (2.04%) *rpoB* mutants were also detected. Three RIF resistant isolates did not show any of MTBDR*plus* assay incorporated *rpoB* mutations; however, wild types WT3/WT4/WT7 were missing. WT8 missing was repeatedly seen in isolates with S531L mutation (73.5%). All of the 52 MTBDR*plus* INH resistant isolates had S315T1 *katG* mutation and WT missing in *katG* at 315 codon. None of the isolates had a mutation in the *inh*A promoter region. Overall, S531L and S315T1 were the two most frequently associated mutants with RIF and INH resistance, respectively. Mutation patterns of INH and RIF drug-resistant *M. tuberculosis* clinical isolates are presented in Table 2.

**Table 2:** Mutations associated with INH and RIF drug resistant *M. tuberculosis* clinical isolates

Phenotypic DST	RIF susceptibility pattern		Phenotypic DST	INH susceptibility pattern					
	*RpoB* gene			*KatG* gene		*InhA* gene			
	WT 1/8	Mutant		WT	Mutant	WT1	WT2	Mutant	Frequency
R	530–533 (WT8)	S531L	R	315	S315T1	–	–	–	35
R	510–513 (WT2)	S531L	R	315	S315T1	–	–	–	1
R	516–519 (WT4)	S531L	R	315	S315T1	–	–	–	2
R	513–517 (WT3) 516–519 (WT4)	D516V	R	315	S315T1	–	–	–	2
R	513–517 (WT3) 516–519 (WT4)	–	R	315	S315T1	–	–	–	1
R	513–517 (WT3) 516–519 (WT4) 530–533 (WT8)	S531L	R	315	S315T1	–	–	–	1
R	526–529 (WT7)	–	R	315	S315T1	–	–	–	2
R	526–529 (WT7)	H526Y	R	315	S315T1	–	–	–	2
R	526–529 (WT7)	S531L	R	315	S315T1	–	–	–	1
R	526–529 (WT7)	H526Y	R	315	S315T1	–	–	–	1
R	–	H526Y	R^a^	–	–	–	–	–	1
S	–	–	R	315	S315T1	–	–	–	4
S	–	–	R^a^	–	–	–	–	–	1

^a^MTBDR*plus* INH susceptible and INH resistant by phenotypic DST

### Discussion

In our study, MTBDR*plus* had the sensitivities of 96.3, 100 and 97.96% in detecting INH resistance, RIF resistance and MDR, respectively and had 100% specificity for each resistance types (INH, RIF, and MDR). The sensitivity of INH resistance detection was comparable with studies reported from Africa; Ethiopia (91.7 and 91.7%, *p* > 0.05) [12, 16], Uganda (88%, *p* > 0.05) [8] and South Africa (94.2 and 85.7%, *p* > 0.05) [9, 11]. However, lower INH sensitivity (80.8 and 62.07%, *p* < 0.05) were also reported by other studies in Uganda [10] and South Africa [11], respectively. The MTBDR*plus* assay was unable to detect two INH resistant *M. tuberculosis* isolates in the routine testing. These discordant cases and lower sensitivity in some areas could be due to a rare mutation that was not incorporated in the MTBDR*plus* assay strips or by mutations in other genomic loci of the *KatG* and *inhA* genes [17].

The sensitivity of detecting RIF resistance of MTBDR*plus* was similar to the report from Ethiopia (100 and 88.2%, *p* > 0.05) [16], Uganda (100%, *p* > 0.05) [10], South Africa (98.9 and 100%, *p* > 0.05) [9, 18]. However, fairly low RIF resistance sensitivity was reported in South Africa (85.7%, *p* < 0.05) [11], and MTB strains with mutations outside of the 81 base-pair region of *rpoB* could be more predominant in that specific study setting. In this study, 100% of RIF resistant cases were detected using MTBDR*plus* assay in routine testing, and RIF resistance was highly associated with mutations in *rpoB* gene of the assay. This ensures all patients identified as RIF resistance had received appropriate treatment with second-line regimen as per the national programmatic management of drug-resistant TB guidelines [7]. The guideline recommends that all RIF resistant patients should be treated as MDR-TB cases using second-line anti-TB drugs.

The sensitivity in detecting MDR-TB is in agreement with most studies in Africa; Ethiopia (100 and 96.4%, *p* > 0.05) [12, 16], Uganda (92.3%, *p* > 0.05) [10] and South Africa (95.6%, *p* > 0.05) [9]. However, the report from South Africa (84.6%, *p* < 0.05) [19] is slightly different.

In agreement with other studies in Ethiopia [12, 16, 19, 20], we found the most common mutation of *rpoB* gene at codon 531 (81.6% of RIF resistant cases) and *katG* gene at codon 315 (100% of INH resistant cases) for RIF and INH resistance, respectively. Target gene mutation analysis can be considered as a drug-resistant testing option in the Ethiopian context.

In general, MTBDR*plus* assay had good routine testing performance in detecting resistance to INH, RIF, and MDR-TB of MTB in comparing against conventional LJ based DST at the hospital level. No single patient was erroneously classified as RIF resistant/MDR and received second-line regimen because of the assay limitation and/or technical incompetence. The MTBDR*plus* assay can be considered to be implemented in hospital settings as far as the safety requirements are fulfilled.

### Limitations

The study was conducted on the stored *M. tuberculosis* isolates. Few of the stored isolates did not grow following subculture and their phenotypic susceptibility test result was unavailable to compare against the MTBDR*plus* assay.

## Abbreviations

ALIP: Aklilu Lemma Institute of Pathology
DST: drug susceptibility testing
EPHI: Ethiopian Public Health Institute
GC: Gregorian calendar
ICA: immunochromatograpic assay
INH: isoniazid
LJ: Lowenstein Jensen
MDR-TB: multidrug resistant TB
MTBC: *Mycobacterium tuberculosis* complex
NPV: negative predictive value
NRL: National TB Reference Laboratory
PCR: polymerase chain reaction
PPV: positive predictive value
RIF: rifampicin
TB: tuberculosis
WHO: World Health Organization
